# Optimizing monocyte-derived immune cell cultures: comparing xeno-free and xenogeneic conditions

**DOI:** 10.3389/fimmu.2025.1589553

**Published:** 2025-10-08

**Authors:** Nora Marek, Anette S. B. Wolff, Harsh N. Dongre, Salwa Suliman

**Affiliations:** ^1^ Center for Translational Oral Research (TOR), Department of Clinical Dentistry, University of Bergen, Bergen, Norway; ^2^ Endocrine Medicine, Department of Clinical Science, University of Bergen, Bergen, Norway; ^3^ Department of Medicine, Haukeland University Hospital, Bergen, Norway; ^4^ Center for Cancer Biomarkers (CCBIO) and Gade Laboratory of Pathology, Department of Clinical Medicine, University of Bergen, Bergen, Norway

**Keywords:** dendritic cell, human AB serum, fetal bovine serum, macrophage, spectral flow cytometry

## Abstract

Culture conditions significantly affect the phenotype of immune cells. This study compared, for the first time, the impact of xeno-free human AB serum and xenogeneic fetal bovine serum (FBS) culture conditions on the surface marker expression of monocyte-derived macrophages (Mo-Mø) and dendritic cells (Mo-DC) using spectral flow cytometry. Monocytes were differentiated into macrophages or Mo-DC over 6 days. M0 macrophages were polarized toward M1-like or M2-like macrophages, and Mo-DC were activated using LPS. Differentiation was successful in both conditions. Despite cells exhibiting autofluorescence, distinct phenotypes based on selected differentiation markers were observed. CD1a expression was lacking in AB cultures, while expression of CD16, CD163 and the co-stimulatory molecules CD80 were significantly upregulated and CD86 downregulated by xenogeneic FBS conditions. These novel findings highlight the need for careful selection of serum type and phenotyping markers to minimize unexpected results and account for potential serum-induced interference with marker expressions in monocyte-derived immune cells.

## Introduction

The immune system consists of innate immunity which provides an unspecific but rapid response and the adaptive immune system that carries out targeted responses. They work together to protect the body from infections while also supporting processes in tissue development, homeostasis and regeneration (1). Adaptive immunity facilitated by T and B lymphocytes requires activation by antigen presenting cells (APC), such as dendritic cells, macrophages or certain B lymphocytes (2). The *in vitro* differentiation of monocytes into monocyte-derived dendritic cells (Mo-DC) or macrophages (Mo-Mø) is well established under specific culture conditions, which typically involve supplementation with animal-derived serum, such as fetal bovine serum (FBS) (3–5). Despite being the most commonly used supplement for culture media, it remains xenogeneic. FBS is typically heat-inactivated to minimize interactions with the complement system of cultured immune cells. Nevertheless, FBS may still contain animal-derived proteins that can trigger immune cell activation. This activation can lead to variability and inconsistency in experimental results observed between studies and can complicate identification of cell phenotypes based on established markers. Therefore, to address these challenges, optimizing culture conditions using xeno-free conditions is crucial to ensuring consistent and reliable results.

Dendritic cells (DC) are the most potent APC to induce adaptive immunity. Their isolation from blood is limited due to low abundance of DC in peripheral blood and challenges related to isolating different DC subsets owing to lack of specific markers (6, 7). *In vivo*, DC differentiate from various precursors that are either myeloid or lymphoid progenitors and give rise to several DC subsets (8). A subset of DC, called inflammatory DC, differentiate from monocytes in tissue during inflammatory conditions (3). Most *in vitro* studies use monocytes as myeloid precursors for DC instead of isolating DC directly from blood or tissues. Macrophages, another innate immune cell type, are naturally differentiated from monocytes upon entering tissues. Monocytes are abundant in peripheral blood and easily isolated via several methods, such as tissue-culture plate adherence or magnetic beads (9). CD14 and CD16 are common markers to isolate and phenotype monocytes, making them attractive precursors for studies of DC activation and macrophages polarization *in vitro*. Monocytes *in vivo* exist in three subsets: classical CD14^+^CD16^–^, intermediate CD14^+^CD16^+^ and non-classical CD14^–^CD16^+^ monocytes (8).

CD14 and CD1 are markers to characterize differentiation of monocytes to DC. CD1 is a group of lipid antigen presenting molecules with five isoforms (CD1a, CD1b, CD1c, CD1e and CD1d). In culture, CD1a, CD1b and CD1c are upregulated upon differentiation into DC while CD14 is downregulated (4). Other markers for characterizing Mo-DC are high expression of MHC-I and II, CD11b, CD11c and low or no expression of FcgRI (CD64) and FCgRIII (CD16) (5). After differentiation, Mo-DC remain in an immature state (5), where generally, immature dendritic cells exert high phagocytic potential with low capacity to stimulate T cell responses (4). The maturation of DC is marked by a simultaneous decrease in phagocytic capacity, and an increase in the expression of stimulatory molecules, including MHC-II, CD80, CD83, CD86 and CD40 (4).

Due to their shared progenitor *in vitro*, many of the described markers such as CD14, MHC-I and II, CD11b and CD11c, used for phenotyping monocytes and DC can also be used to identify monocyte-derived macrophages (Mo-Mø) (10). For classically activated macrophages (M1), upregulation of stimulatory molecules MHC-II, CD80 and CD86 is observed. Non-classically activated macrophages (M2) are generally marked by CD163 and CD206 expression (10–12).

Most studies comparing culture conditions for myeloid cells primarily focus on Mo-Mø and the cytokines used for their differentiation (12–14). Macrophages are typically differentiated using macrophage colony-stimulating factor (M-CSF) or granulocyte-macrophage colony-stimulating factor (GM-CSF), whereas Mo-DC are differentiated with GM-CSF and IL-4. Former studies have examined the expression profiles of Mo-Mø cultured with M-CSF, GM-CSF or a combination of both, with most relying on FBS as a culture supplement (12, 13, 15). As there are various methods for generating monocyte-derived immune cells, comparing studies and identifying reliable surface markers can be challenging. These issues underscore the need for culturing immune cells in xeno-free media to ensure reproducibility and translational relevance of findings. To our knowledge, no study to date has compared the use of xeno-free culture conditions with xenogeneic (FBS-based) conditions to characterize diverse myeloid cells using spectral flow cytometry. This lack of data hinders the interpretation of results.

**Figure 1:**
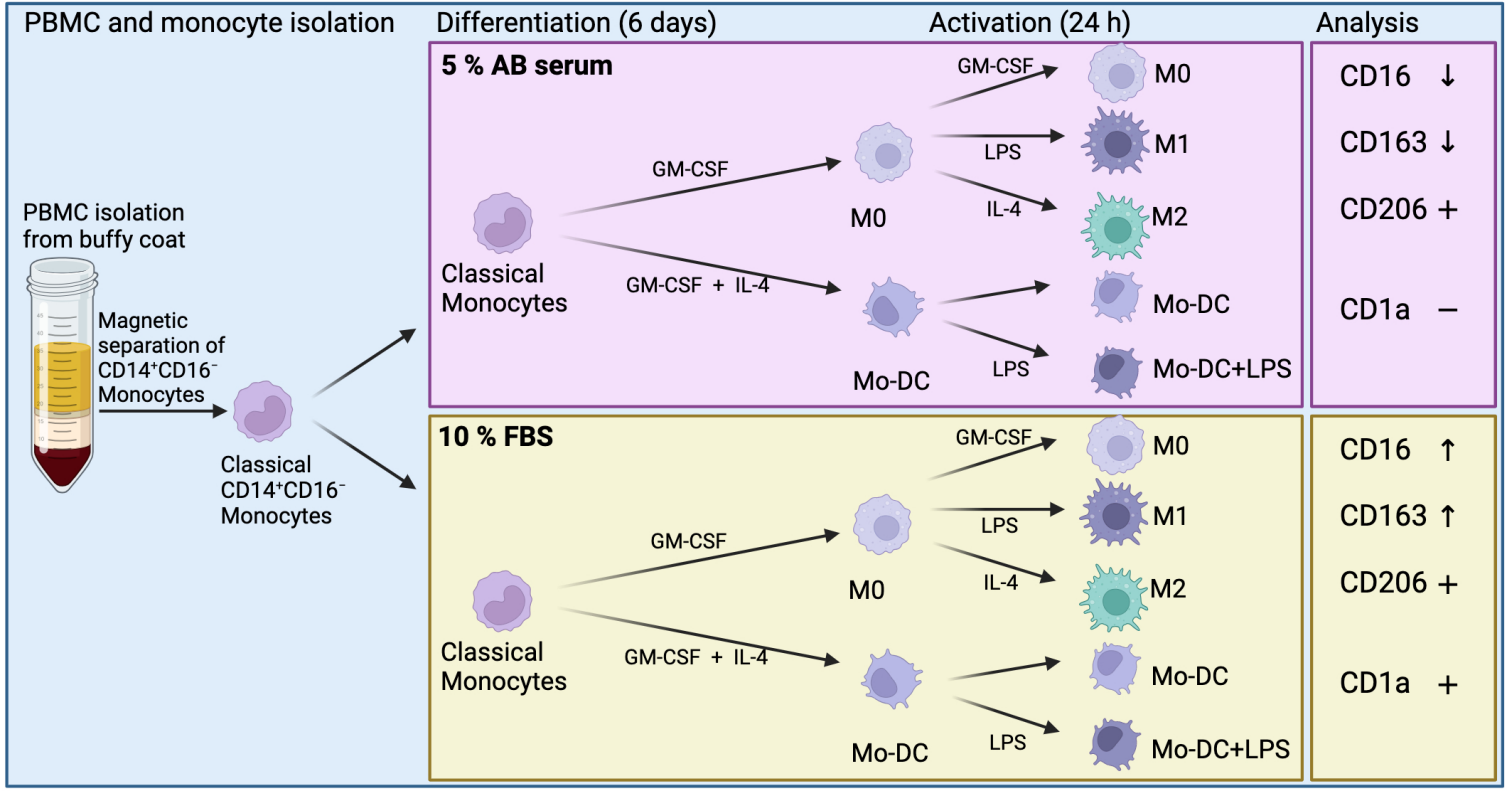
Schematic overview of methods and results of monocyte-derived dendritic cells (Mo-DC) and monocyte-derived macrophages. Differentially expressed surface markers in relation to each culture condition (CD16 and CD163), and overall expression of surface markers (CD1a and CD206). Created in BioRender. Suliman, S. (2025) https://BioRender.com/mgfey7n.

This study assessed the effects of xenogeneic (FBS) or xeno-free (human AB serum) culture conditions on the phenotype and activation status of human monocyte-derived immune cells using spectral flow cytometry, aiming to enhance detection sensitivity and improve the reliability of result comparisons. A schematic overview of the study is illustrated in **Figure 1**.

## Methods

### Classical monocyte isolation and cell culture

Buffy coat preparations were obtained from healthy donors (N = 3) after consent from the Blood Bank, Haukeland University Hospital, Bergen (Dok-ID AIT-70729). Donors were from both male and female and of different ages. Peripheral blood mononuclear cells (PBMC) were isolated using Ficoll density gradient following a standard PBMC isolation protocol. Subsequently, CD14^+^CD16^–^ monocytes were isolated by magnetically activated cell sorting (MACS) using the Classical Monocyte Isolation Kit following the manufacturer’s manual (Miltenyi, Germany). Unlabeled monocytes were counted and seeded in RPMI supplemented with 5% human AB serum (Sigma-Aldrich/Merck, Germany) or 10% heat-inactivated FBS (HyClone, GE Healthcare, USA) and 1% Penicillin/Streptomycin (Thermo Fisher Scientific, USA) with a cell density of 2×10^5^ - 2.5×10^5^ cells/cm^2^ in standard tissue culture plates. FBS was heat-inactivated for 30 min at 56 °C while mixing regularly (16).

### Classical monocyte differentiation and activation

To induce differentiation into Mo-DC, the medium was supplemented with 50 ng/mL GM-CSF (R&D, USA) and 20 ng/mL IL-4 (Miltenyi, Germany), while non-activated macrophages (M0) were cultured with 20 ng/mL GM-CSF. The medium was changed every three days by removing half the volume and replenishing it with cytokine-supplemented medium. On day 6 of culture, 100 ng/mL Lipopolysaccharide (LPS) was added to Mo-DC cultures to stimulate activation, while M0 were activated for 24 h with 100 ng/mL LPS or 20 ng/mL IL-4 to induce M1 or M2, respectively.

### Immunophenotyping by spectral flow cytometry

To analyze the phenotypical differences of cells cultured in human serum or FBS, cells were collected on day 7. Briefly, 0.5-1×10^6^ cells were stained with Fixable Live/Dead (Invitrogen, USA) for 30 min at 4°C protected from light. After washing, unspecific binding was blocked with 2 µL of 2% normal mouse serum (Invitrogen, USA) in 100 µL MACS buffer (Miltenyi, Germany) for 15 min at room temperature protected from light. Antibodies targeting extracellular antigens (**Supplementary Table S1**) (all from Invitrogen, USA) in MACS buffer containing Super Bright (SB) complete staining buffer (Invitrogen, USA) were added directly into blocking solution and incubated for 30 min at 4°C protected from light. Cells were washed and fixed using Intracellular (IC) Fixation Buffer (Invitrogen, USA) for 30 min at room temperature protected from light. Fixed cells were washed in 1× Permeabilization buffer (Perm buffer; Invitrogen, USA) and intracellular staining for CD68 was carried out in 1× Perm buffer for 30 min at room temperature protected from light. Stained cells and unstained controls were run in MACS buffer using a Sony ID7000™ Spectral Cell Analyzer (Sony Biotechnology, USA) equipped with 5 lasers (355 nm, 405 nm, 488 nm, 561 nm, 637 nm). The similarity matrix for each fluorophore given in **Supplementary Table S1** can be found in **Supplementary Figure S1A**. The gating strategy is shown in **Supplementary Figure S2**.

### Data analysis and statistical analysis

The flow data was unmixed and autofluorescence was removed using the Sony ID7000™ software (v1.1.10). Doublets and dead cells were excluded from the analysis. Plots and mean fluorescence intensity were prepared using the Sony ID7000™ software (v1.1.10), while uniform manifold approximation and projection for dimension reduction (uMap) were prepared using FlowJo™ (v10 software and later, BD Life Sciences, USA). Statistical analysis was conducted by using Two-way ANOVA with *ad-hoc* test in GraphPad (version 5 and later, California, USA). Data are expressed as the mean ± standard error of the mean (SEM). Differences were considered statistically significant at p <0.05. Mo-DC and Mo-Mø were differentiated from the same donor in each experimental set-up, and the experiments were repeated using 3 different donors.

## Results

### Impact of macrophage autofluorescence on unmixing

The myeloid cells, particularly macrophages, exhibited strong autofluorescence when cultured in tissue culture plates for 7 days. The autofluorescence spectrum was consistent among macrophage phenotypes (M0 and M1 depicted, **Supplementary Figure S1B**). Autofluorescence intensities above 10^3^ were considered high, with the strongest signals observed in the 400–500 nm range, excited by 355 nm (UV), 405 nm (V) and 488 nm (B) lasers. This autofluorescence spectrum overlapped with certain fluorophores commonly used in flow cytometry. Notably, there was significant overlap between eFluor506 and the autofluorescence spectrum (**Supplementary Figure S1C**, yellow arrow), leading to false negatives when unmixing autofluorescence for eFluor506. (**Supplementary Figure S1C**, yellow arrow). To improve this, the antibody conjugated to eFluor506 was replaced with FITC in subsequent experiments, as FITC exhibited less spectral overlap with the autofluorescence, thereby improving unmixing.

### Impact of different serum on lineage markers on both Mo-DC and Mo-Mø

**Figure 2:**
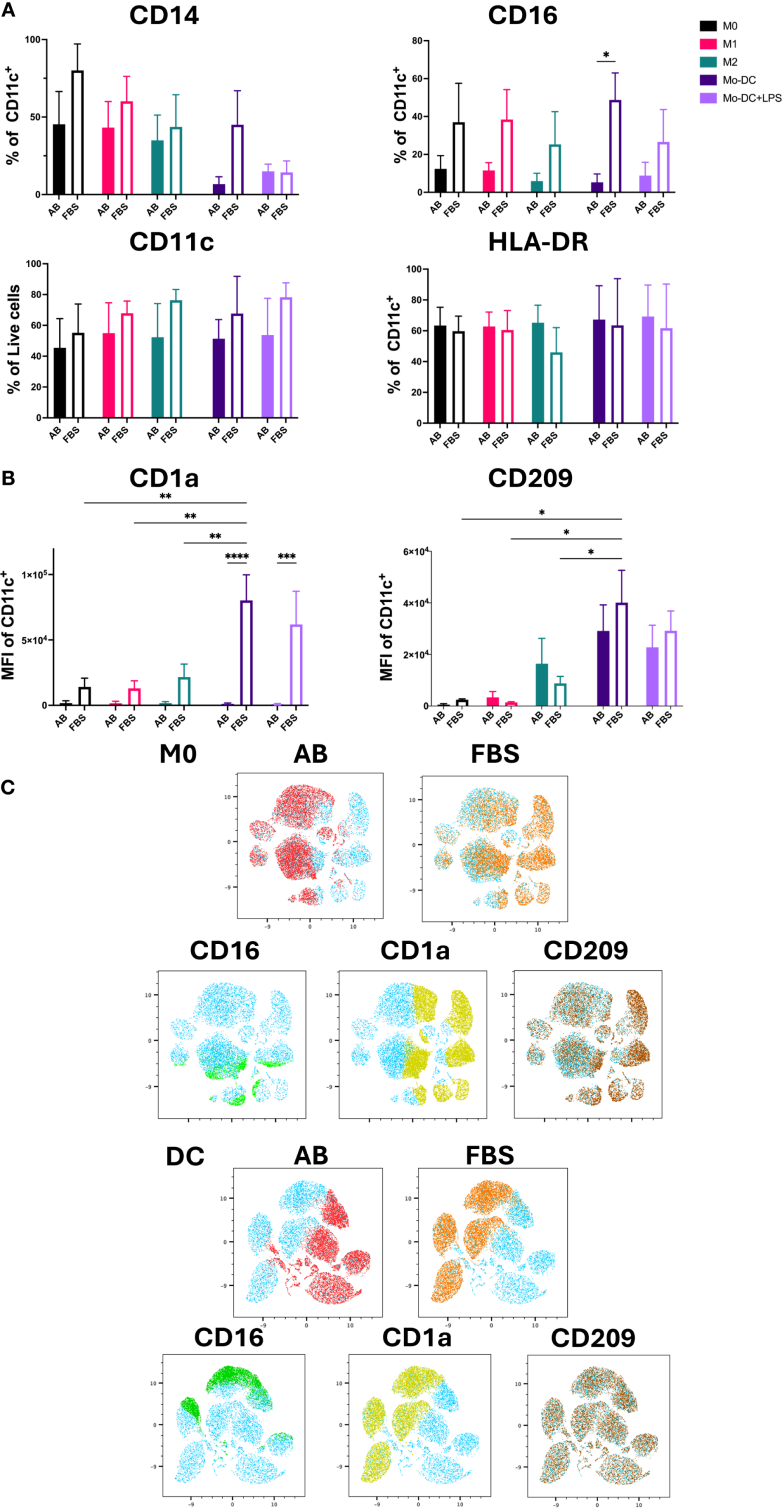
Mo-DC lineage markers on both Mo-Mø and Mo-DC. **(A)** Quantitative expression of lineage surface markers averaged from 3 donors. Two-way ANOVA and *post-hoc* test: *p<0.05. **(B)** MFI of Mo-DC lineage surface markers (CD1a and CD209) averaged from 3 donors. Two-way ANOVA and *post-hoc* test: *p<0.05, **p<0.01, ***p<0.001, ****p<0.0001. **(C)** uMap showing localization of surface markers CD16 (green), CD1a (yellow) and CD209 (brown) of M0 and Mo-DC in AB (red) or FBS (orange).

Previous reports have shown that the markers expressed by isolated monocytes, including CD14, CD68, CD11c and HLA-DR, should be expressed by all Mo-Mø phenotypes, while Mo-DC should retain the expression of the monocyte markers CD11c, HLA-DR and CD68 (5, 7, 8). Our results showed that in both culture conditions these markers were expressed on non-activated M0 and activated M1 and M2 macrophages, while Mo-DC expressed CD68 (data not shown), CD11c, HLA-DR and had mostly downregulated CD14 (**Supplementary Figure S3A**; **Figure 2A**). Therefore, successful differentiation from classical monocytes was evidenced by Mo-Mø expressing CD14, CD68, CD11c and HLA-DR, while Mo-DC exhibiting CD11c and HLA-DR with downregulated CD14. CD16 was expressed by Mo-DC and Mo-Mø during culture in AB serum and FBS, with significantly increased expression (p<0.05) observed in FBS cultured cells (**Figure 2A**).

CD209 and CD1a are commonly used lineage markers for differentiating Mo-DC (17, 18). Differentiated Mo-DC portrayed a significant upregulation of CD209 expression (>90%) under both serum culture conditions compared to M0 and M1 (p<0.01 and p<0.001, respectively) cultured with either AB or FBS (**Supplementary Figure S3B**). Additionally, the mean fluorescence intensity (MFI) of CD209 in Mo-DC was significantly increased compared to Mo-Mø cultured with FBS. While a slight, non-significant upregulation of CD209 expression and MFI was observed in M2 macrophages compared to M0 and M1, this difference was minimal (**Figure 2B**; **Supplementary Figure S3B**). Significant differences in CD1a MFI were observed between AB and FBS culture conditions for Mo-DC and Mo-DC+LPS (p<0.0001 and p<0.001, respectively; **Figure 2B**). In Mo-DC cultured with FBS, CD1a MFI was significantly higher compared to Mo-Mø (p<0.01; **Figure 2B**). Similarly, to MFI, CD1a expression levels were significantly upregulated in FBS culture conditions when comparing Mo-DC and Mo-Mø to those cultured with AB culture conditions (p<0.001 and p<0.0001; **Supplementary Figure S3B**). For Mo-DC, a significant increase in CD1a expression was observed only in FBS culture conditions when comparing Mo-DC to M1 or M2 (p<0.05, p<0.01 and p<0.001; **Supplementary Figure S3B**).

The correlation of marker expression was visualized using uMap, highlighting the localization of CD1a, CD16 and CD209 expression in M0 and Mo-DC cultured with AB and FBS (**Figure 2C**).

### Impact of different serum on maturation markers of Mo-DC and activation markers of Mo-Mø

**Figure 3:**
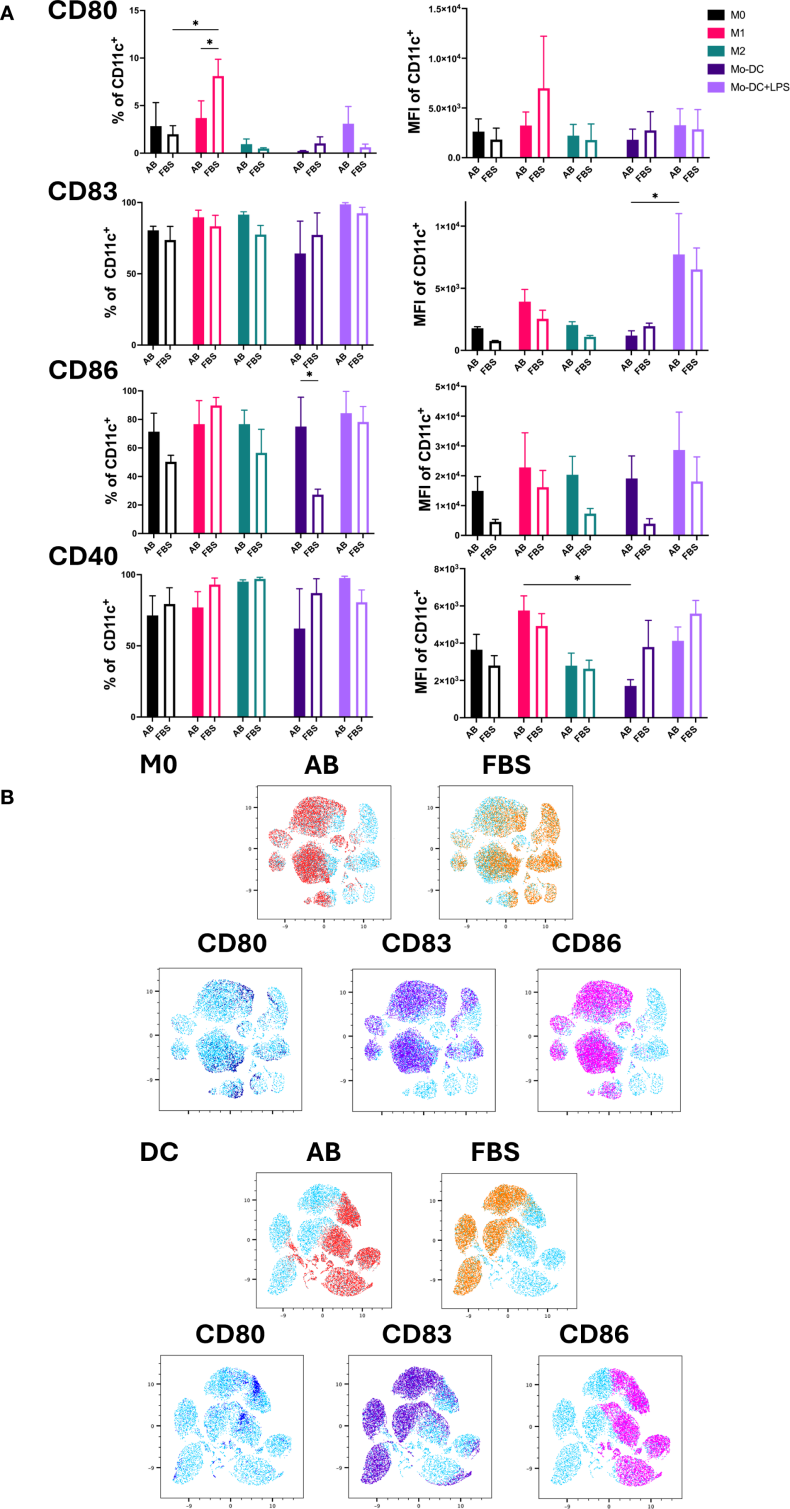
Activation markers of Mo-Mø and Mo-DC. **(A)** Quantitative expression and MFI of surface activation markers CD80, CD83, CD86 and CD40 of Mo-Mø and Mo-DC averaged from 3 donors. Two-way ANOVA and *post-hoc* test: *p<0.05. **(B)** uMap showing localization of surface activation markers CD80 (blue), CD83 (purple) and CD86 (pink) of M0 and Mo-DC in AB (red) or FBS (orange).

The expression of the co-stimulatory molecules CD80, CD83, CD86 and CD40 was used as an indicator of DC maturation and Mo-Mø activation (**Figure 3A**) (3, 18, 19). LPS stimulated Mo-DC exhibited increased expression of CD80, CD83 and CD86 under both AB and FBS culture conditions. The expression of CD86 was higher in Mo-DC cultured with AB compared to FBS (p<0.05). CD83 expression was significantly upregulated in Mo-DC+LPS compared to Mo-DC in AB culture conditions (p<0.05; **Figure 3A**). Overall, CD80 expression in Mo-DC+LPS was low under both AB and FBS culture conditions, and only a slight increase of CD40 was observed (**Figure 3A**).

In Mo-Mø, expression of the co-stimulatory molecules CD83, CD86 and CD40 was relatively high (>50%) for both culture conditions. A significant upregulation of CD80 was observed in M1 cultured with FBS compared to M0 (p<0.05; **Figure 3A**). Additionally, M1 showed higher CD80 expression in FBS culture conditions compared to AB (p<0.05; **Figure 3A**).

The correlation of marker expression was visualized using uMap, demonstrating the localization of CD80, CD83 and CD86 in M0 and Mo-DC cultured with AB and FBS (**Figure 3B**).

### Impact of different serum on polarization markers of activated Mo-Mø: M1-like and M2-like

**Figure 4:**
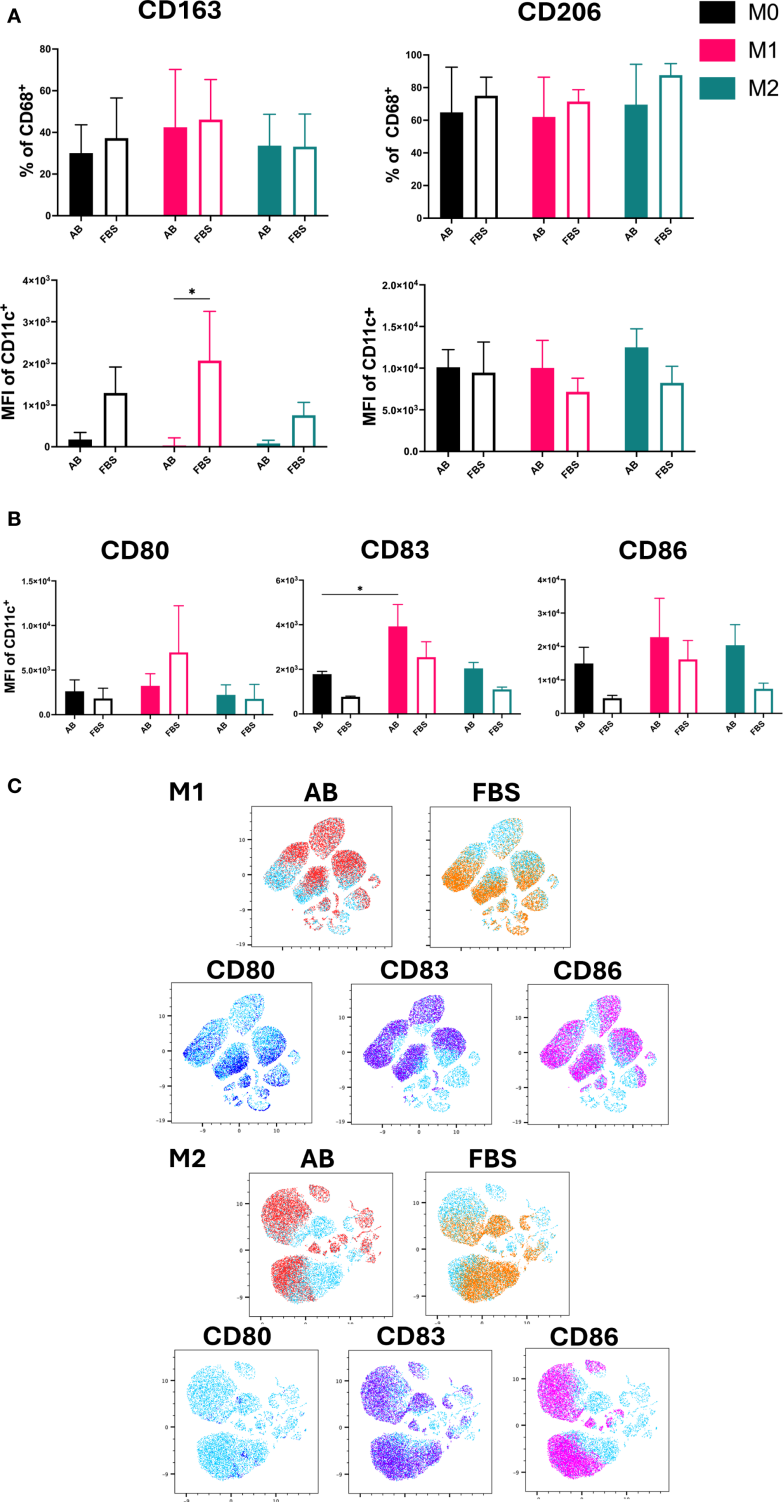
M1/M2 Polarization markers of Mo-Mø. **(A)** Quantitative expression and MFI of surface polarization markers CD163 and CD206 for Mo-Mø (M0, M1 and M2) averaged from 3 donors. Two-way ANOVA and *post-hoc* test: *p<0.05. **(B)** Mean fluorescence intensity (MFI) of surface activation markers CD80, CD83 and CD86 for Mo-Mø (M0, M1 and M2) averaged from 3 donors. Two-way ANOVA and *post-hoc* test: *p<0.05. **(C)** uMap showing localization of surface activation markers CD80 (blue), CD83 (purple) and CD86 (pink) of M1 and M2 macrophages in AB (red) or FBS (orange).

The expression and MFI of CD163 and CD206 were used as markers for M2-like macrophages (**Figure 4A**), while the MFI of co-stimulatory molecules CD80, CD83 and CD86 was used to identify M1-like macrophages (**Figure 4B**) (19, 20). Both M2 markers, CD163 and CD206, were expressed by all Mo-Mø.

Differences between culture conditions were only observed for the MFI of CD163. In M1 cultured with FBS, a significant increase in the MFI of CD163 was observed (p<0.05; **Figure 4A**). Although a slight increase in CD163 MFI was noted in M0 and M2 cultured with FBS, this was not statistically significant. No clear trends were observed for CD206.

For M1, the MFI of CD83 was significantly higher compared to M0 in AB culture conditions (p<0.05; **Figure 4B**). Additionally, an upregulation of CD86 was observed in M1 and M2 compared to M0 in both serum culture conditions, albeit the increase was not significant.

The correlation of marker expression was visualized using uMap and the localization of CD80, CD83 and CD86 was demonstrated in M1 and M2 cultured with AB and FBS (**Figure 4C**).

## Discussion

Due to the potential of xenogeneic factors activating innate immune cells, we aimed to compare the expression of commonly used surface markers for immune cell phenotyping on monocyte-derived immune cells cultured in xenogeneic (FBS) and xeno-free (human AB serum) serum conditions. Our results showed that conventional surface markers, CD1a and CD163, were of limited utility to discern differentiation and polarization state of monocyte-derived immune cells using xeno-free culture conditions. Monocyte-derived cells exhibit strong autofluorescence, particularly in the ultraviolet, violet and blue-green channels, which can pose significant challenges for unmixing spectral flow cytometry data. This autofluorescence can produce signals as high as 10^4^ in unstained samples, as observed for eFluor506, FITC and SB436. While culture conditions, including the use of AB or FBS serum, and the activation status of the cells can influence the intensity of autofluorescence, the autofluorescence spectrum itself is expected to remain conserved. This inherent autofluorescence underscores the need for careful selection of fluorophores to minimize spectral overlap and ensure accurate data interpretation.

CD80, a costimulatory activation marker for antigen presenting cells, particularly in M1-like macrophages, was upregulated under both serum conditions tested. However, a significantly higher expression was observed in cultures supplemented with FBS compared to AB. These findings align with previous observations reporting increased MFI of CD80 in M1 macrophages cultured in FBS relative to those in human platelet lysate, with a distinction from M2 macrophage profile (21). Although the referenced study differs in several aspects, such as the use of M-CSF, and different human serum sources; it still supports the trend observed here. Therefore, we speculate that FBS may promote a more pro-inflammatory macrophage phenotype. This could influence not only *in vitro* experimental design, but also the development of macrophage-based therapeutic strategies and their downstream effects on T cell activation.

As for M2-like macrophages, although CD163 and CD206 are widely referenced in the literature as markers for M2-like macrophages, our findings, along with results from other studies, did not show significant differences in the surface expression of these markers between M0, M1-like and M2-like subsets (12, 20, 22). These observations suggest that CD163 and CD206 may not reliably distinguish M2-like macrophages based solely on surface expression levels under certain experimental conditions. Classical monocytes already have high expression of CD163, which was also observed for our monocytes (data not shown) (23, 24). Flow cytometry data can be presented using various parameters for analysis beyond percentage expression (%), such as absolute mean fluorescence intensity (MFI) or representation as ratios. These different parameters are particularly useful for detecting subtle changes in marker expression that may not have been evident through percentage expressions alone. Previous reports showed changes in CD163 and CD206 expression between M1-like and M2-like macrophages through MFI only. These changes were discernible (9, 10, 12, 22), which makes interpretations more robust. In our MFI data, we also showed a change in CD163 expression between polarization states, however, the data demonstrated through percentage expressions showed no differences. This again highlights how different parameters are useful to detect minute changes in specific markers that facilitate data comparisons. Ratios have been reported to represent data and show differences (14). In some reports when data is only represented as ratios, valuable information including absolute expression percentage and absolute fluorescence intensity of the compared phenotypes and their control are commonly not published. This complicates data interpretation and comparison between studies. Therefore, in this study focused on phenotype characterization we chose to present both absolute expression and absolute fluorescence intensity.

Besides serum, other culture supplements such as cytokines can also affect expression on macrophages. CD163 was shown to be only upregulated with IL-10, not other anti-inflammatory cytokines IL-4 and IL-13 (24). Our study used IL-4 to polarize Mo-Mø into M2-like macrophages, which may explain a lack of significant upregulation of CD163. CD163 is a scavenger receptor, primary involved in the clearance of hemoglobin complexes, but has also been shown to recognize bacteria. It functions as an innate immune sensor on tissue-resident macrophages and contributes to the regulation of local immune responses (25). Based on our observations, we speculate that extended culture of monocytes on tissue culture-treated plates may promote the acquisition of a tissue-like macrophage phenotype, including upregulation of CD163, reflecting aspects of *in vivo* differentiation. Differentiation into Mo-DC was successfully achieved in our experiments under both AB or FBS culture conditions, as indicated by the high expression of CD209. We used GM-CSF for differentiation of monocytes for Mo-Mø, and GM-CSF and IL-4 for Mo-DC. Interestingly, we also observed CD209 expression on macrophages, possibly due to GM-CSF in the culture condition as GM-CSF can induce expression of CD209 (26). IL-4 has been found to be the main cytokine that induces DC differentiation and with it CD209 expression.

GM-CSF has been shown to have additive effects on CD209 expression together with IL-4 (26). Therefore, GM-CSF or IL-4 alone induced low expression of CD209, however, combined they strongly induce CD209 expression, which was observed in our results on Mo-DC and Mo-Mø (26). We observed that when inducing M2 phenotypes with IL-4, CD209 was further upregulated compared to M0 and M1 regardless of culture conditions, consistent with findings reported by other groups (10, 15). This suggests that CD209 expression may not be exclusive to Mo-DC and could reflect the influence of specific cytokine environments on macrophage populations. CD209, by mediating the uptake of mannose-rich motifs and promoting T cell adhesion via ICAM-3, plays a crucial role in the adaptive immune response (27). Thus, upregulation of CD209 on DC or macrophages may enhance antigen presentation and facilitate T cell activation. This has important biological implications, as it may influence the immune response. Therefore, in co-culture experiments modeling immune activation or host-pathogen interactions, CD209 expression should be carefully considered when designing and interpreting experiments. CD206, another phagocytosis receptor recognizing mannose-rich glycoproteins, has also been reported to be upregulated in Mo-Mø cultured in GM-CSF (28, 29). It has roles in host defense and has been linked to anti-inflammatory responses (hence used as a marker for M2-like responses) and given importance for wound healing and tissue repair and resolution of inflammation (30, 31). This expression was consistent with our data, as we also observed high expression of CD206 in all Mo-Mø, with highest trend in M2-like macrophages albeit insignificant. This highlights our previous discussion that CD206 might not be a reliable marker for M2-like macrophages. GM-CSF differentiation does not only affect the surface markers, but also cytokine release, Itoh and colleagues have shown that GM-CSF cultured Mo-Mø have reduced IL-10 production (29). This highlights the importance of utilizing complementary analytical approaches to gain a comprehensive understanding of marker expression, and culture-dependent phenotype distinctions in monocyte-derived cells.

A strong correlation was observed between CD1a expression, another commonly used marker for Mo-DC differentiation, and FBS culture conditions. Both Mo-Mø and Mo-DC cultured in FBS exhibited a pronounced upregulation in CD1a, while expression levels in AB cultured cells remained low in both cell types. This finding aligns with studies by Lehner et al. and others, which reported a lack of CD1a expression in Mo-DC cultured in media containing increasing percentages of human serum (32–34). CD1a is a lipid antigen-presenting molecule, predominantly expressed by Langerhans cells and associated with mucosal and skin immunity (35, 36). It presents lipid antigen to CD1a-restricted T cells and is known to be upregulated during inflammatory conditions (35). As such, CD1a may serve as an indicator of enhanced T cell activation and CD1a driven responses (35, 36). However, the underlying mechanisms of lipid antigen presentation to CD1a-restricted T cells, including the identity of relevant lipid antigens and the mode of T cell activation, remain unraveled. These uncertainties are particularly relevant in co-culture systems aiming to study lipid antigen presentation or gamma delta T cell responses. Our findings suggest that serum, and especially the use of FBS, significantly influences CD1a expression. Therefore, serum conditions and nonetheless percentage concentrations should be heeded. These results highlight a critical finding: CD1a might not be a reliable marker for Mo-DC differentiation in AB culture conditions. Notably, FBS induced CD1a expression on both Mo-Mø and Mo-DC, potentially leading to misinterpretation of different outcomes. Thus, researchers using xeno-free cell cultures need to be aware of these differences to avoid prematurely discarding or misinterpreting results.

Our study used primary cells, which inherently introduce donor variability, and can complicate the interpretation of results. As such, there is a critical need for more reliable markers to distinguish between monocyte-derived cell phenotypes, enabling robust conclusions. While investigating gene transcription and protein release would provide complementary information about cellular behavior, we chose to focus on surface markers analyzed by spectral flow cytometry. This approach was selected because surface markers offer a more reliable, efficient, and well-established method for determining immune cell phenotypes rapidly, particularly in the context of comparative studies. Nevertheless, consistent trends of expression were observed in the three independent donors analyzed (both male and female of a wide age range). This highlights the strength and reproducibility of the markers chosen and validated in this comparative study. Another strength in our design is the ability of spectral flow cytometry to accommodate comprehensive panels. This approach adds significant value by allowing detailed analysis of co-expression patterns in monocyte-derived cells, making it possible for us to investigate both Mo-Mø and Mo-DC within the same sample in a single run.

While this study provides valuable insights, a few limitations should be acknowledged to appropriately interpret the findings. As this report focused solely on spectral flow cytometry, we could not assess protein secretion or transcriptional changes induced by the different serum conditions. For studies exploring immune responses to pathogens, cancer, or therapeutics, additional methods are needed to confirm macrophage polarization or Mo-DC differentiation and activation. Functional assays, particularly those investigating Mo-DC-induced T cell responses in co-cultures, are essential (33). However, conditions used for co-culturing should be well-defined in advance; an aspect we specifically aimed to optimize in this study. Our findings are specific to the polarization protocols used in this study, which although commonly used, are not the only approaches available for inducing distinct phenotypes in macrophages. While we deliberately used a single cytokine per condition to avoid confounding effects during differentiation, this simplified approach may also be considered a limitation. Selecting appropriate polarization and activation controls, such as cytokine combinations or alternative agonists (37) should align with the research question. In our study, LPS was used for M1 and IL-4 for M2 polarization; however, more robust or distinct activation states might be achieved with combined stimuli.

We also acknowledge that the study was limited to *in vitro* analysis of monocyte-derived immune cells, reducing the system complexity. Although *in vivo* models offer broader physiological relevance, they may not directly reflect human biology. Recent advances in complex *in vitro* systems, including 3D (38, 39) and *in silico* models (40), offer promising alternatives but rely heavily on foundational data from simplified *in vitro* systems. Detailed knowledge of cell behavior defined *in vitro* settings, such as those presented here, is essential for developing and interpreting these complex models. Such systems hold promises for identifying disease-specific immune biomarkers and stimulating immune responses to cell or drug therapies in a precision medicine context (38–40).

Notably, our findings also point to a gap in current knowledge regarding the biological functions commonly used *in vitro* markers. This underlines the need for continued investigation into monocyte-derived cells to better understand the relevance and regulation of differentially expressed surface markers under standardized conditions.

Taken together, our findings could ultimately help researchers in making more informed and standardized decisions in experimental designs when either using AB or FBS serum. Our results have shown distinct differences in surface marker expressions between monocyte-derived immune cells cultured in AB or FBS serum. M1/M2 markers CD163 and CD206 are highly dependent on culture conditions. CD209 remains a robust marker for Mo-DC differentiation, although expression on macrophages may require cautious interpretation. The variability of CD1a expression across different serum conditions calls for a re-evaluation of its utility as a universal marker for Mo-DC, particularly in human-based systems. This highlights the importance of carefully choosing the type of serum used to culture these cells in relation to the experimental hypothesis sought and analytical methods used. Also, considering marker validation to ensure accurate and reproducible results in immune cell studies.

## Data Availability

The raw data supporting the conclusions of this article will be made available by the authors, without undue reservation.

## Supplementary material

Supplementary Figure 1
**(A)** Similarity matrix of used panel. **(B)** Representative autofluorescence spectra of unstained Mo-Mø (shown M0 and M1). **(C)** Spectral overlap of autofluorescence with eFluor506 (yellow arrow) and FITC, and data spread after unmixing (yellow arrow).

Supplementary Figure 2
**(A)** Gating strategy for Mo-DC stain and **(B)** Mo-Mø stain.

Supplementary Figure 3
**(A)** Expression and MFI of CD68 on Mo-Mø. N = 3. Mean ± SEM. **(B)** Expression of Mo-DC markers CD1a and CD209 on Mo-Mø and Mo-DC. Averaged from 3 independent donors Mean ± SEM. 2way ANOVA and *post hoc*. ** p<0.01, *** p< 0.001, **** p<0.0001.

## References

[1] Rankin LC Artis D. Beyond host defense: emerging functions of the immune system in regulating complex tissue physiology. Cell. (2018) 173:554–67. doi: 10.1016/j.cell.2018.03.013, PMID: 29677509

[2] Marshall JS Warrington R Watson W Kim HL. An introduction to immunology and immunopathology. Allergy Asthma Clin Immunol. (2018) 14:49. doi: 10.1186/s13223-018-0278-1, PMID: 30263032 PMC6156898

[3] Randolph GJ Inaba K Robbiani DF Steinman RM Muller WA. Differentiation of phagocytic monocytes into lymph node dendritic cells *in vivo* . Immunity. (1999) 11:753–61. doi: 10.1016/S1074-7613(00)80149-1, PMID: 10626897

[4] Sallusto F Lanzavecchia A. Efficient presentation of soluble antigen by cultured human dendritic cells is maintained by granulocyte/macrophage colony-stimulating factor plus interleukin 4 and downregulated by tumor necrosis factor alpha. J Exp Med. (1994) 179:1109–18. doi: 10.1084/jem.179.4.1109, PMID: 8145033 PMC2191432

[5] Romani N Gruner S Brang D Kämpgen E Lenz A Trockenbacher B. Proliferating dendritic cell progenitors in human blood. J Exp Med. (1994) 180:83–93. doi: 10.1084/jem.180.1.83, PMID: 8006603 PMC2191538

[6] Sanarico N Ciaramella A Sacchi A Bernasconi D Bossù P Mariani F. Human monocyte-derived dendritic cells differentiated in the presence of il-2 produce proinflammatory cytokines and prime th1 immune response. J Leukocyte Biol. (2006) 80:555–62. doi: 10.1189/jlb.1105690, PMID: 16809642

[7] Flórez-Grau G Escalona JC Lacasta-Mambo H Roelofs D Bödder J Beuk R. Human dendritic cell subset isolation by magnetic bead sorting: A protocol to efficiently obtain pure populations. Bio Protoc. (2023) 13:e4851. doi: 10.21769/BioProtoc.4851, PMID: 37900109 PMC10603258

[8] Ziegler-Heitbrock L Ancuta P Crowe S Dalod M Grau V Hart DN. Nomenclature of monocytes and dendritic cells in blood. Blood. (2010) 116:e74–80. doi: 10.1182/blood-2010-02-258558, PMID: 20628149

[9] Nielsen MC Andersen MN Møller HJ. Monocyte isolation techniques significantly impact the phenotype of both isolated monocytes and derived macrophages *in vitro* . Immunology. (2020) 159:63–74. doi: 10.1111/imm.13125, PMID: 31573680 PMC6904589

[10] Buchacher T Ohradanova-Repic A Stockinger H Fischer MB Weber V. M2 polarization of human macrophages favors survival of the intracellular pathogen chlamydia pneumoniae. PloS One. (2015) 10:e0143593. doi: 10.1371/journal.pone.0143593, PMID: 26606059 PMC4659546

[11] Jaguin M Houlbert N Fardel O Lecureur V. Polarization profiles of human M-csf-generated macrophages and comparison of M1-markers in classically activated macrophages from gm-csf and M-csf origin. Cell Immunol. (2013) 281:51–61. doi: 10.1016/j.cellimm.2013.01.010, PMID: 23454681

[12] Hickman E Smyth T Cobos-Uribe C Immormino R Rebuli ME Moran T. Expanded characterization of *in vitro* polarized M0, M1, and M2 human monocyte-derived macrophages: bioenergetic and secreted mediator profiles. PloS One. (2023) 18:e0279037. doi: 10.1371/journal.pone.0279037, PMID: 36862675 PMC9980743

[13] Lacey DC Achuthan A Fleetwood AJ Dinh H Roiniotis J Scholz GM. Defining gm-csf– and macrophage-csf–dependent macrophage responses by *in vitro* models. J Immunol. (2012) 188:5752–65. doi: 10.4049/jimmunol.1103426, PMID: 22547697

[14] Lukic A Larssen P Fauland A Samuelsson B Wheelock CE Gabrielsson S. Gm-csf– and M-csf–primed macrophages present similar resolving but distinct inflammatory lipid mediator signatures. FASEB J. (2017) 31:4370–81. doi: 10.1096/fj.201700319R, PMID: 28637652

[15] Sander J Schmidt SV Cirovic B McGovern N Papantonopoulou O Hardt A-L. Cellular differentiation of human monocytes is regulated by time-dependent interleukin-4 signaling and the transcriptional regulator ncor2. Immunity. (2017) 47:1051–66.e12. doi: 10.1016/j.immuni.2017.11.024, PMID: 29262348 PMC5772172

[16] Urzì O Bergqvist M Lässer C Moschetti M Johansson J D´Arrigo D. Heat inactivation of foetal bovine serum performed after ev-depletion influences the proteome of cell-derived extracellular vesicles. J Extracell Vesicles. (2024) 13:e12408. doi: 10.1002/jev2.12408, PMID: 38263378 PMC10805629

[17] Chometon TQ Siqueira M Sant´anna JC Almeida MR Gandini M Martins de Almeida Nogueira AC. A protocol for rapid monocyte isolation and generation of singular human monocyte-derived dendritic cells. PloS One. (2020) 15:e0231132. doi: 10.1371/journal.pone.0231132, PMID: 32271804 PMC7145147

[18] Sánchez-Torres C García-Romo GS Cornejo-Cortés MA Rivas-Carvalho A Sánchez-Schmitz G. Cd16+ and cd16– human blood monocyte subsets differentiate *in vitro* to dendritic cells with different abilities to stimulate cd4+ T cells. Int Immunol. (2001) 13:1571–81. doi: 10.1093/intimm/13.12.1571, PMID: 11717198

[19] Nicod LP Joudrier S Isler P Spiliopoulos A Pache J-C. Upregulation of cd40, cd80, cd83 or cd86 on alveolar macrophages after lung transplantation. J Heart Lung Transplant. (2005) 24:1067–75. doi: 10.1016/j.healun.2004.07.011, PMID: 16102442

[20] Bertani FR Mozetic P Fioramonti M Iuliani M Ribelli G Pantano F. Classification of M1/M2-polarized human macrophages by label-free hyperspectral reflectance confocal microscopy and multivariate analysis. Sci Rep. (2017) 7:8965. doi: 10.1038/s41598-017-08121-8, PMID: 28827726 PMC5566322

[21] Lopa S Libonati F Mareschi K Talò G Brambilla S Raffo V. Using macrophage polarization in human platelet lysate to test the immunomodulatory potential of cells for clinical use. Biomedicines. (2024) 12(4):833. doi: 10.3390/biomedicines12040833, PMID: 38672188 PMC11048141

[22] Unuvar Purcu D Korkmaz A Gunalp S Helvaci DG Erdal Y Dogan Y. Effect of stimulation time on the expression of human macrophage polarization markers. PloS One. (2022) 17:e0265196. doi: 10.1371/journal.pone.0265196, PMID: 35286356 PMC8920204

[23] Parfieniuk-Kowerda A Grubczak K Eljaszewicz A Świderska M Maciaszek M Panasiuk A. High cd163 expression on classical monocytes is associated with immune control of hbv infection in noncirrhotic patients. Mediators Inflammation. (2020) 2020:6364258. doi: 10.1155/2020/6364258

[24] Sulahian TH Högger P Wahner AE Wardwell K Goulding NJ Sorg C. Human monocytes express cd163, which is upregulated by il-10 and identical to P155. Cytokine. (2000) 12:1312–21. doi: 10.1006/cyto.2000.0720, PMID: 10975989

[25] Fabriek BO van Bruggen R Deng DM Ligtenberg AJM Nazmi K Schornagel K. The macrophage scavenger receptor cd163 functions as an innate immune sensor for bacteria. Blood. (2009) 113:887–92. doi: 10.1182/blood-2008-07-167064, PMID: 18849484

[26] Relloso M Puig-Kröger A Pello O Rodríguez-Fernández JL de la Rosa G Longo N. Dc-sign (Cd209) expression is il-4 dependent and is negatively regulated by ifn, tgf-B, and anti-inflammatory agents1. J Immunol. (2002) 168:2634–43. doi: 10.4049/jimmunol.168.6.2634, PMID: 11884427

[27] Engering A Geijtenbeek TBH van Vliet SJ Wijers M van Liempt E Demaurex N. The dendritic cell-specific adhesion receptor dc-sign internalizes antigen for presentation to T cells1. J Immunol. (2002) 168:2118–26. doi: 10.4049/jimmunol.168.5.2118, PMID: 11859097

[28] Bender AT Ostenson CL Giordano D Beavo JA. Differentiation of human monocytes *in vitro* with granulocyte–macrophage colony-stimulating factor and macrophage colony-stimulating factor produces distinct changes in cgmp phosphodiesterase expression. Cell Signalling. (2004) 16:365–74. doi: 10.1016/j.cellsig.2003.08.009, PMID: 14687666

[29] Itoh CY Gunnarsson C Babunovic GH Nibasumba A Akilimali NA Wadsworth MH. Gm-csf differentiation of human monocytes stabilizes macrophage state via oxidative signaling. bioRxiv. (2020). doi: 10.1101/2020.09.29.318352. 2020.09.29.318352.

[30] Martinez-Pomares L. The mannose receptor. J Leukocyte Biol. (2012) 92:1177–86. doi: 10.1189/jlb.0512231, PMID: 22966131

[31] Tsuchiya K Suzuki Y Yoshimura K Yasui H Karayama M Hozumi H. Macrophage mannose receptor cd206 predicts prognosis in community-acquired pneumonia. Sci Rep. (2019) 9:18750. doi: 10.1038/s41598-019-55289-2, PMID: 31822747 PMC6904766

[32] Jakobsen MA Møller BK Lillevang ST. Serum concentration of the growth medium markedly affects monocyte-derived dendritic cells’ Phenotype, cytokine production profile and capacities to stimulate in mlr. Scand J Immunol. (2004) 60:584–91. doi: 10.1111/j.0300-9475.2004.01515.x, PMID: 15584969

[33] Lehner M Morhart P Stilper A Holter W. Functional characterization of monocyte-derived dendritic cells generated under serumfree culture conditions. Immunol Lett. (2005) 99:209–16. doi: 10.1016/j.imlet.2005.02.016, PMID: 16009271

[34] Švajger U. Human platelet lysate is a successful alternative serum supplement for propagation of monocyte-derived dendritic cells. Cytotherapy. (2017) 19:486–99. doi: 10.1016/j.jcyt.2017.01.005, PMID: 28215928

[35] de Jong A Ogg G. Cd1a function in human skin disease. Mol Immunol. (2021) 130:14–9. doi: 10.1016/j.molimm.2020.12.006, PMID: 33348245 PMC8549081

[36] Wegrecki M. Cd1a-mediated immunity from a molecular perspective. Mol Immunol. (2023) 158:43–53. doi: 10.1016/j.molimm.2023.04.010, PMID: 37116273

[37] Murray PJ Allen JE Biswas SK Fisher EA Gilroy DW Goerdt S. Macrophage activation and polarization: nomenclature and experimental guidelines. Immunity. (2014) 41:14–20. doi: 10.1016/j.immuni.2014.06.008, PMID: 25035950 PMC4123412

[38] Hammel JH Cook SR Belanger MC Munson JM Pompano RR. Modeling immunity *in vitro*: slices, chips, and engineered tissues. Annu Rev BioMed Eng. (2021) 23:461–91. doi: 10.1146/annurev-bioeng-082420-124920, PMID: 33872520 PMC8277680

[39] Miserocchi G Bocchini M Cortesi M Arienti C De Vita A Liverani C. Combining preclinical tools and models to unravel tumor complexity: jump into the next dimension. Front Immunol. (2023) 14 - 2023:1171141. doi: 10.3389/fimmu.2023.1171141, PMID: 37033986 PMC10080004

[40] Palsson S Hickling TP Bradshaw-Pierce EL Zager M Jooss K O’Brien PJ. The development of a fully-integrated immune response model (Firm) simulator of the immune response through integration of multiple subset models. BMC Syst Biol. (2013) 7:95. doi: 10.1186/1752-0509-7-95, PMID: 24074340 PMC3853972

