# Supplementary figures and images for: Optimizing monocyte-derived immune cell cultures: comparing xeno-free and xenogeneic conditions
Source: Front Immunol. 2025 Oct 8;16:1589553. doi: 10.3389/fimmu.2025.1589553 (PMC12540148; doi:10.3389/fimmu.2025.1589553)

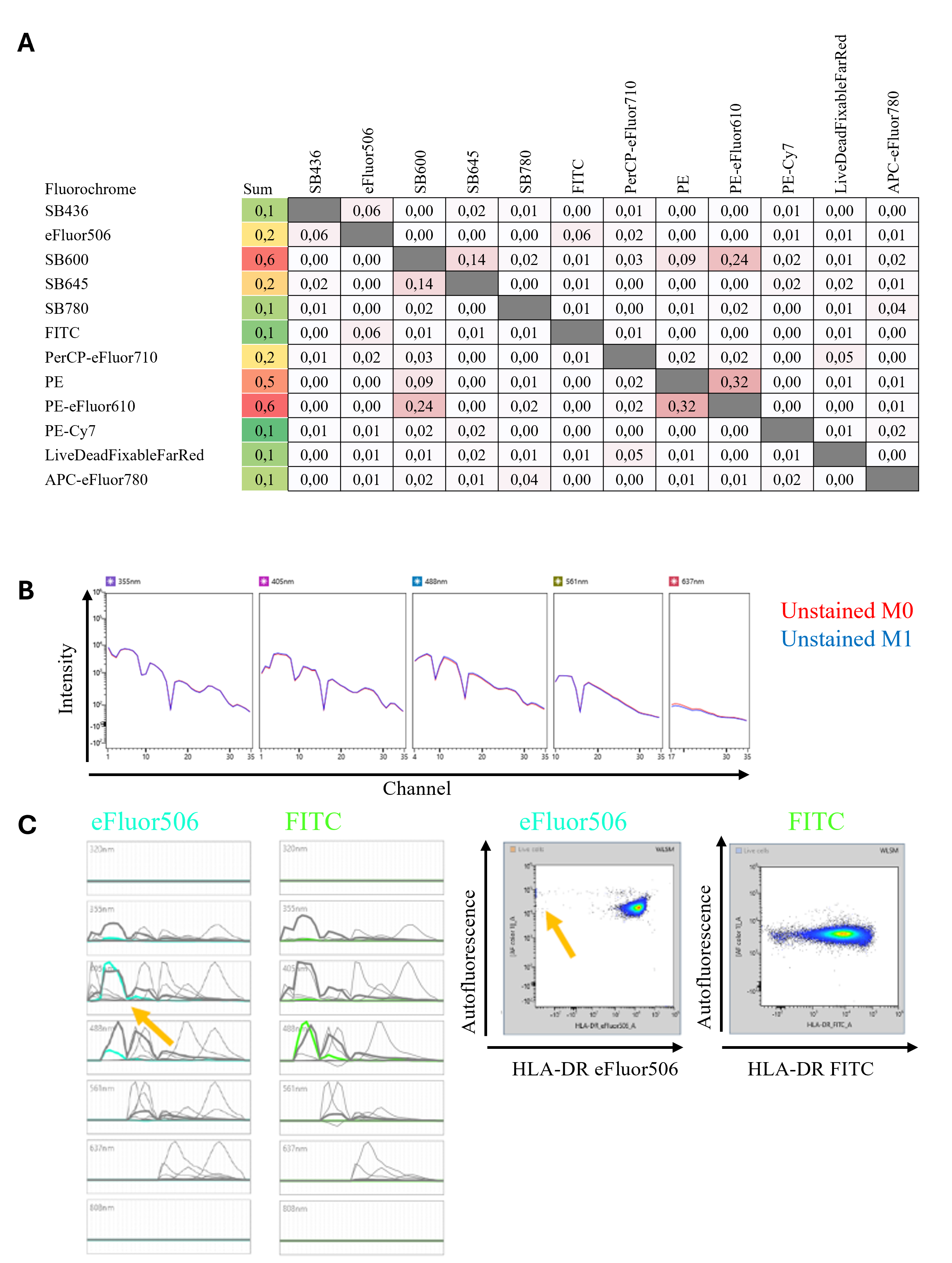

Supplement: Supplementary Figure 1 — (A) Similarity matrix of used panel. (B) Representative autofluorescence spectra of unstained Mo-Mø (shown M0 and M1). (C) Spectral overlap of autofluorescence with eFluor506 (yellow arrow) and FITC, and data spread after unmixing (yellow arrow). [file Image1.tif]

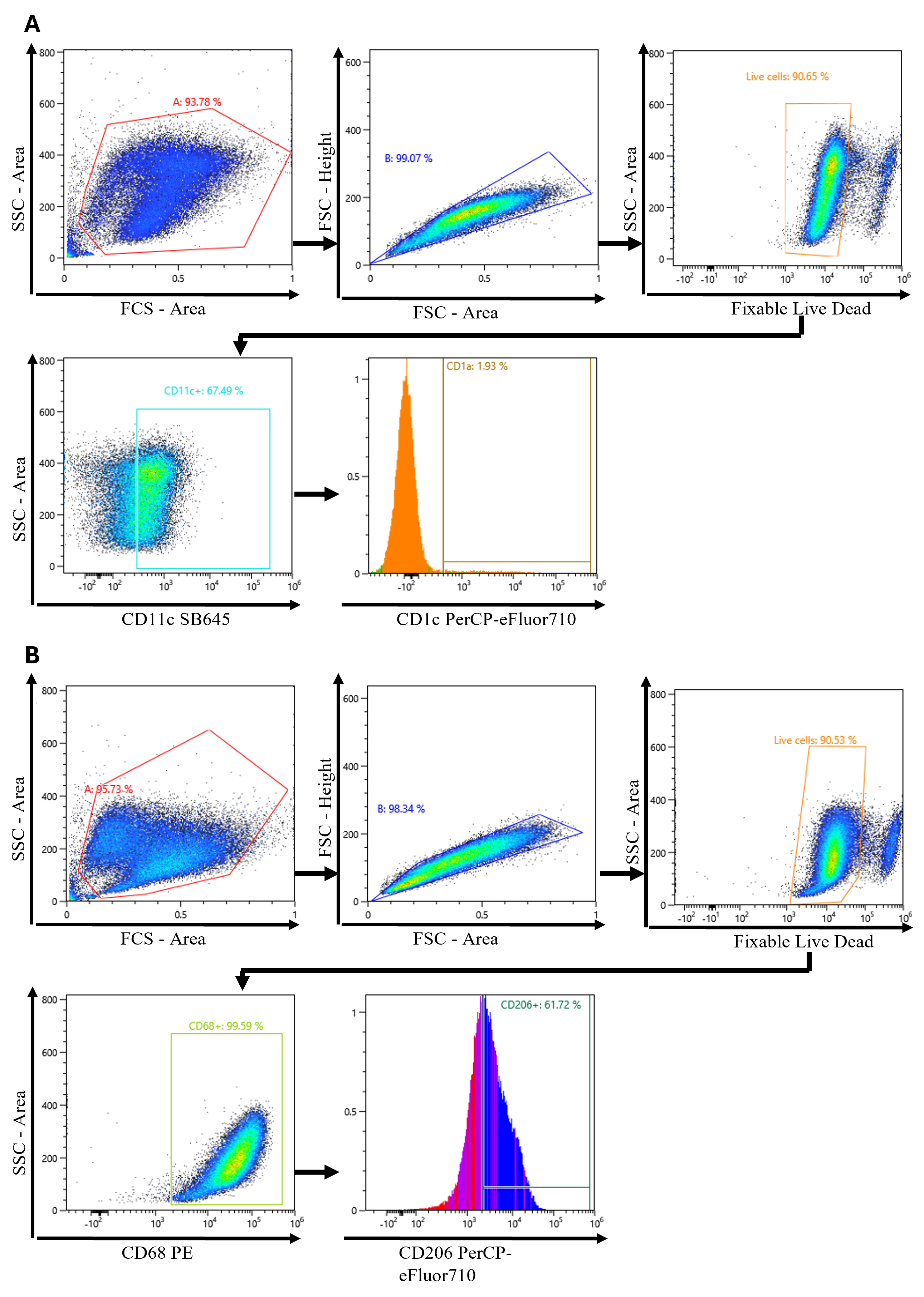

Supplement: Supplementary Figure 2 — (A) Gating strategy for Mo-DC stain and (B) Mo-Mø stain. [file Image2.tif]

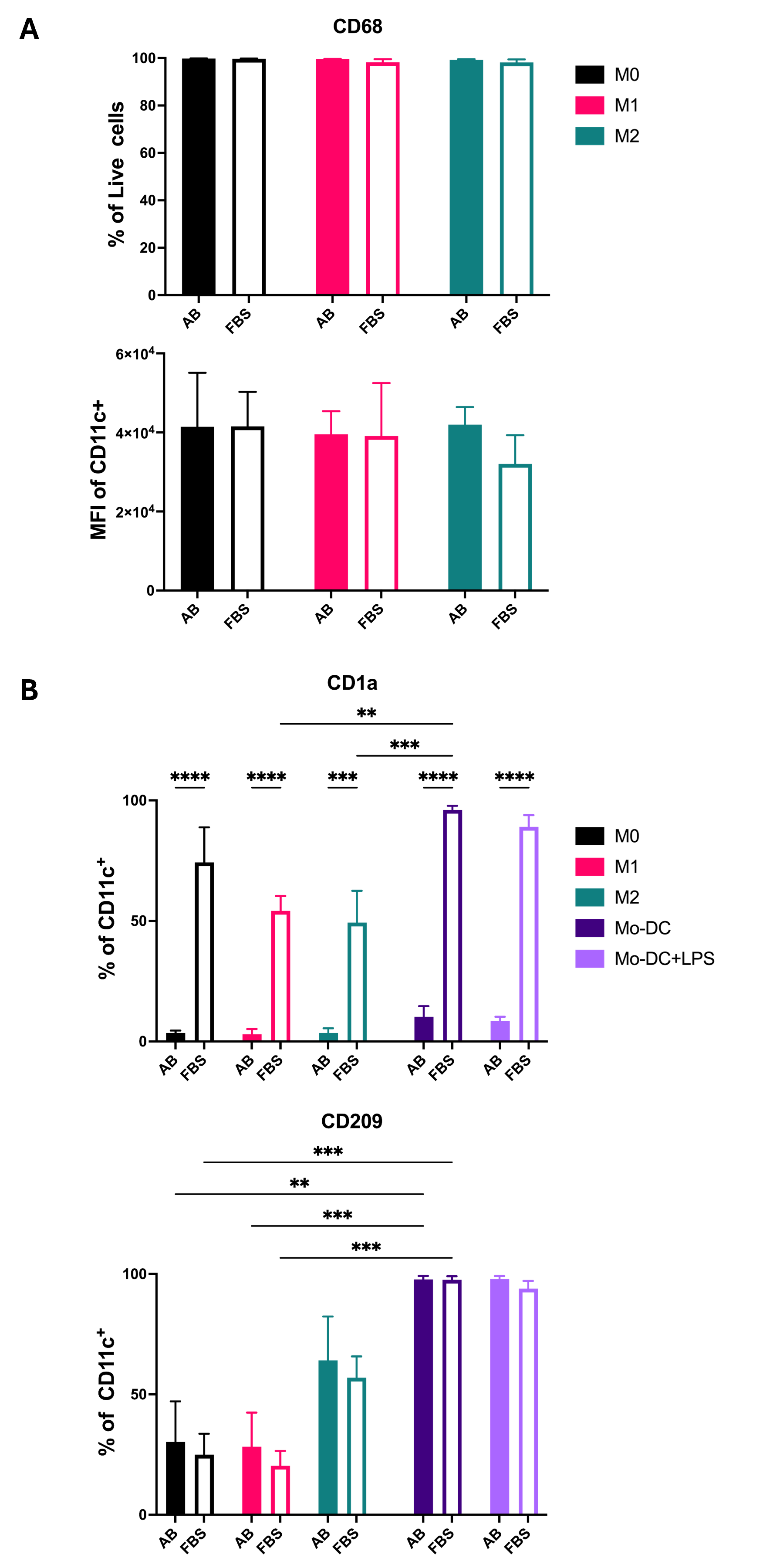

Supplement: Supplementary Figure 3 — (A) Expression and MFI of CD68 on Mo-Mø. N = 3. Mean ± SEM. (B) Expression of Mo-DC markers CD1a and CD209 on Mo-Mø and Mo-DC. Averaged from 3 independent donors Mean ± SEM. 2way ANOVA and post hoc. ** p<0.01, *** p< 0.001, **** p<0.0001. [file Image3.tif]
