# Supplementary material for: Optimizing monocyte-derived immune cell cultures: comparing xeno-free and xenogeneic conditions
Source: Front Immunol. 2025 Oct 8;16:1589553. doi: 10.3389/fimmu.2025.1589553 (PMC12540148; doi:10.3389/fimmu.2025.1589553)
Supplement: Supplementary file 4 [file Table1.docx]

**Supplementary Table 1:** Antibody information and dilution in MACS buffer used for immune cell staining

| **Antibody Fluorophore (Clone)** | **Macrophage** | **Dendritic cell** |
| --- | --- | --- |
| LIVE/DEAD™ Fixable Far Red Dead Cell Stain Kit | 1:2000-3000 | 1:2000-3000 |
| Anti-CD11c SB645 (3.9) | 1:50 | 1:50 |
| Anti-CD1a PercP-eFluor710 (HI149) | - | 1:50 |
| Anti-CD14 PE-eFluor610 (61D3) | 1:100 | 1:100 |
| Anti-CD16 SB780 (eBioCB16 (CB16)) | 1:100 | 1:100 |
| Anti-HLA-DR eFluor506 or FITC (LN3) | 1: 600 | 1: 600 |
| Anti-CD80 SB436 (2D10.4) | 1:100 | 1:100 |
| Anti-CD86 SB600 (IT2.2) | 1:200 | 1:200 |
| Anti-CD83 PE-Cy7 (HB15e) | - | 1:100 |
| Anti-CD40 APC-eFluor 780 (5C3) | 1:100 | 1:100 |
| Anti-CD163 PE-Cy7 (eBioGHI/61 (GHI/61)) | 1:100 | - |
| Anti-CD206 PercP-eFluor710 (19.2) | 1:100 | - |
| Anti- CD209 PE (eB-h209) | - | 1: 600 |
| Anti-CD68 PE (eBioY1/82A (Y1/82A)) | 1: 600 | - |
